# Feasibility, acceptability, and preliminary effectiveness of the adapted Namaste Care program delivered by caregivers of community-dwelling older persons with moderate to advanced dementia: a mixed methods feasibility study

**DOI:** 10.1186/s12877-022-03483-9

**Published:** 2022-10-13

**Authors:** Marie-Lee Yous, Jenny Ploeg, Sharon Kaasalainen, Carrie McAiney, Kathryn Fisher

**Affiliations:** 1 School of Nursing, McMaster University, 1280 Main Street West, Hamilton, ON L8S 4K1 Canada; 2 School of Public Health Sciences, Schlegel Research Chair in Dementia, Schlegel-UW Research Institute for Aging, University of Waterloo, 200 University Avenue West, Waterloo, ON N2L 3G1 Canada; 3 School of Nursing, Aging, Community and Health Research Unit, Faculty of Health Sciences, McMaster University, 1280 Main Street West, Room HSc3N25, Hamilton, ON L8S 4K1 Canada

**Keywords:** Dementia, Caregivers, Family, Older Persons, Namaste Care, Community, Psychosocial Intervention, Mixed Methods, Quantitative, Qualitative

## Abstract

**Background:**

Caregivers have considerable responsibilities in supporting persons in advanced stages of dementia, however they receive little education. Namaste Care is a multisensory program originally designed to be delivered by healthcare providers in long-term care homes for persons with advanced dementia. The program has not yet been adapted and evaluated for use by caregivers of persons with moderate to advanced dementia living at home. The purpose of this feasibility study is to determine the feasibility, acceptability and preliminary effectiveness of the adapted Namaste Care program for use by caregivers of community-dwelling older persons with moderate to advanced dementia.

**Methods:**

This feasibility study, with a one-group before-after design and interviews, was part of a larger study using a multiphase mixed methods design. A total of 12 caregivers delivered the program over three months. Caregivers completed questionnaires on caregiver quality of life, perceptions of caregiving, self-efficacy, and burden at baseline and 3-month follow-up. Caregivers participated in interviews at the 3-month follow-up to explore acceptability and perceived benefit. Descriptive statistics and paired t-tests were used to analyze quantitative data. A secondary analysis used multiple imputation to explore the impact of missing data. Experiential thematic analysis was used in analyzing qualitative data.

**Results:**

The adapted Namaste Care program was judged to be feasible, given that all caregivers used it at least twice a week over the 3-month period. The retention rate of caregivers was 83% (10 of 12). Caregivers perceived that the program was practical, enhanced the wellbeing of persons with dementia, and brought them closer in their relationships with persons with dementia. There were no statistically significant changes for quality of life, perceptions of caregiving, self-efficacy, or burden outcomes. Multiple imputation results revealed promising findings for an improvement in caregiver wellbeing related to quality of life.

**Conclusions:**

The adapted Namaste Care program for use by caregivers of community-dwelling older persons with moderate to advanced dementia was feasible and acceptable. The program has the potential to enhance the quality of life and other outcomes of caregivers, however there is a need to conduct a larger trial that is adequately powered to detect these effects.

**Supplementary Information:**

The online version contains supplementary material available at 10.1186/s12877-022-03483-9.

## Background

Over 50 million individuals worldwide are living with dementia [1]. The number of people impacted by dementia is expected to double every two decades with a projected increase to 78 million people by 2030 due to a growing global aging population [2]. In Canada, 60–70% of persons with dementia are living at home to remain independent and engaged in their community [3, 4]. Approximately 50% of Canadians with moderate dementia and 14% of persons with advanced dementia live at home rather than a retirement or long-term care (LTC) home [5]. As individuals progress towards moderate to advanced stages of dementia they will experience significant changes such as profound memory loss, minimal speech, loss of independent ambulation, and inability to complete activities of daily living [6]. At this stage, persons with dementia living at home will require greater supports from family and friend caregivers.

Despite the growing numbers of caregivers supporting older persons with dementia at home, most caregivers receive very little education and support in their role. In a sample of 246 caregivers of community-dwelling persons with dementia from the United States, more than 85% of caregivers reported that they had unmet needs related to caregiver education [7 ]. Most of these caregivers were caring for a person with moderate (38%) or advanced (18%) dementia at home [7]. A lack of education and support contributes to caregiver stress, burden, depression, poor physical health and wellbeing, and low confidence levels in caring for older persons with dementia [8]. A solution to meeting the educational needs of caregivers is the implementation of a psychosocial intervention. Many psychosocial interventions are intended for both caregivers and persons with dementia. Psychosocial interventions are intended to slow or prevent a decline in mental and physical health of caregivers as well as persons receiving care by targeting caregiver competencies, knowledge, activities and/or relationships [9].

Psychosocial interventions (e.g., leisure activities and exercise programs, counselling, and support programs) [10] can help caregivers build their confidence in providing meaningful activities that uphold personhood and promote social engagement of persons with dementia [11]. Some psychosocial interventions are intended to be delivered as shared activities between caregivers and persons with dementia. For example, some interventions require that caregivers deliver leisure activities for persons with dementia at home which can lead to decreased caregiver burden [12]. Leisure and physical activity interventions are perceived by caregivers as being enjoyable through co-participation and offering temporary relief of daily tasks which results in improvements in physical and mental health [10]. These interventions have been found to increase the quality of life (QOL) of caregivers, their knowledge of dementia, and skills in delivering care [13–16]. Psychosocial interventions delivered by caregivers are a safe alternative to pharmacological interventions to support the QOL of persons with dementia [17–19]. When persons with dementia are provided with meaningful activities that enhance their QOL this in turn may improve the QOL of caregivers.

Most psychosocial interventions are intended for those with early to moderate dementia [14]. To date there are few psychosocial approaches intended for older persons with moderate to advanced dementia and suitable to be delivered by caregivers at home [20]. Among the studies evaluating psychosocial programs, few report outcomes based on the severity of dementia [21–24]. In addition to the lack of interventions for people with moderate to advanced dementia, caregivers have had little to no involvement in designing psychosocial interventions [22, 25–27]. Their lack of involvement in program design can limit the fit of interventions with their realities of caregiving. Namaste Care is a promising psychosocial intervention that can be used by caregivers of persons with moderate to advanced dementia at home [28]. It has not yet been adapted and evaluated for use by caregivers in a community home setting (e.g., house, condo, apartment). To date, caregivers have not been included in research to adapt Namaste Care so that it can be used in a home setting. In order to address this gap in this study, a version of Namaste Care that was adapted by caregivers for use in their home was evaluated to assess feasibility, acceptability, and preliminary effectiveness.

### Namaste Care

Namaste Care is a psychosocial, multisensory program that was originally developed for use in LTC in the United States to fill a gap related to a lack of programs suitable for persons with advanced dementia [28]. The core principles of Namaste Care consist of creating a comfortable environment and using an unhurried, loving touch approach during interactions with persons with dementia [28]. The theory informing Namaste Care is that the spirit of a person with advanced dementia continues to be present despite profound changes happening to his or her body and mind. The spirit can be nurtured by others through meaningful activities and loving touch [28]. Namaste Care was originally based on a person-centred approach [28] that has since evolved towards relationship-centred care where interactions between individuals are valued as building blocks for therapeutic and meaningful activities [29]. There are multiple dimensions to caring relationships that considers the experiences of individuals giving care such as caregivers and those receiving care such as persons with dementia [29]. Namaste Care combines different modalities including music, massage, reminiscing, socialization, aromatherapy, and snacks. The program provides practical skills for healthcare providers to meaningfully engage people with dementia in activities. For persons with dementia, the goals of the program are to enhance their physical and mental wellbeing, decrease distress and pain, and provide them with meaningful activities to facilitate connections and socialization. For healthcare providers and families, the goals of the program are to equip them with knowledge, skills, and confidence in delivering meaningful activities, support them in recognizing signs of distress and pain in persons with advanced dementia, and enhance their overall wellbeing by being able to form connections with persons with advanced dementia [28].

Namaste Care sessions are delivered in a quiet room with low lighting and no distractions. In LTC settings, the sessions are expected to last 2 h and are delivered daily in the morning and afternoon. People with dementia are welcomed into a designated room where relaxing music is being played in the background and scents (e.g., lavender, seasonal scents) are being diffused. Namaste Care incorporates principles of touch to provide stimulation for persons with dementia. Healthcare providers provide tactile activities throughout the session such as hand/foot massages, applying lotions, and hair brushing. Healthcare providers speak to the person with dementia throughout the session. Persons with dementia are provided with a Namaste Care Toolbox that has unique items based on individual preferences such as lotions, life-like dolls, photos, plush animals, and balls to provide sensory stimulation. Throughout the Namaste Care program persons with dementia are being monitored by healthcare providers for signs of pain and discomfort [28]. Caregivers may participate in the sessions.

The core principles of the Namaste Care program that should remain intact when being adapted include creating a comfortable environment and using an unhurried, loving touch approach. The frequency and duration of the program is tailorable based on the setting of delivery and who is delivering the program. Ideally the program should be delivered daily, however there is no rigid expectation for how often and for how long the program should be delivered. The program can be delivered by more than one individual including more than one family member. Activities that are part of Namaste Care such as scenting a room, playing music, providing touch activities, offering beverages and snacks, encouraging range of motion activities, and providing reminiscence activities (e.g., photo albums, conversations about life stories) are expected to be tailored to the abilities and interests of individuals with dementia [28].

Namaste Care has now been used internationally in various settings including LTC, hospice, acute care, and home settings (where it was delivered by hospice volunteers) [28]. Tasseron-Dries et al. (2021) evaluated Namaste Care in LTC homes in the Netherlands by implementing an adapted version of the original program that placed a greater emphasis on involving families in the program, however only nursing staff and volunteers received formal training [30, 31]. Active family caregiver involvement was hindered in the study as family caregivers were unsure about the purpose of their involvement in the program and its potential benefits [30]. There is only one study that explores the use of Namaste Care in a home setting [32]. In this study, Namaste Care was delivered by volunteers of a hospice in England who went into the homes of persons with dementia. The program was found to increase socialization for persons with dementia [32]. Although the program was implemented in a home setting, the focus was on training volunteers, not caregivers, to deliver the program.

In LTC settings, Namaste Care has resulted in positive changes for persons with dementia such as reduced use of antianxiety medications and other psychotropic medications, lower risk of delirium, lessened pain symptoms, and reduced responsive behaviours while improving QOL and relationships with staff [30, 33–37]. In LTC settings, family members feel more comfortable and relaxed when interacting with persons with dementia when engaging in Namaste Care because they have a better understanding of the progressive nature of dementia, suitable activities, and communication approaches [36]. Improvements for persons with advanced dementia have been found because Namaste Care combines physical, emotional, and multisensory care to increase social connections, reduce depression, and keep them alert and engaged in meaningful activities for longer periods during the day resulting in less anxiety, distress, and need for antipsychotics [33]. Effects of the program for persons with dementia can impact the overall wellbeing of caregivers as well. By being actively engaged in a meaningful activity this can help to address boredom and lead to reduced pain for persons with dementia. Pain is modulated by the cognitive status, emotions, and previous experiences of an individual [38]. Persons with dementia are therefore highly susceptible to pain due to impaired cognition and regulation of emotions.

It is essential to assess feasibility of interventions when considering their delivery in a new setting and when in-depth knowledge of the target population’s environmental and social context was not considered in previous studies [39]. Feasibility studies are used to determine whether an intervention can be implemented and how it should be implemented [40]. Feasibility studies are designed to determine the feasibility of research methods so that they can be repeated in a larger scale study or reveal potential effects that warrant further investigation in a follow-up larger scale study [41]. In terms of program evaluation for caregivers, some researchers have conducted trials to evaluate interventions without first conducting feasibility studies [26, 42]. Assessing feasibility is of particular significance in the current study because Namaste Care has not yet been implemented in a home setting by caregivers and it is unknown whether it will be accepted by caregivers or feasible for them to deliver the program. Findings of the feasibility study can inform whether a larger study such as a randomized controlled trial is warranted and feasible to evaluate effects of the program.

### The adapted Namaste Care program

The Namaste Care program was adapted in terms of content, care activities and implementation process to be used for persons with moderate to advanced dementia. The primary adaptations were reducing the frequency of delivery from twice a day to twice a week, integrating Namaste Care activities into daily activities and routines rather than at scheduled times as done in LTC, and providing a one-hour training session for caregivers with bi-weekly check-ins for opportunities for more resources and education rather than a full day of training. As part of the program, caregivers were provided with a training manual with links to videos, a tailored kit, and bi-weekly individual check-ins. The adaptation process and findings are presented in greater detail elsewhere [43].

Adaptation was informed by conducting small group virtual (i.e., videoconference or phone) workshop sessions with caregivers who had experience in supporting persons living with moderate to advanced dementia at home. A training guide was created, and caregiver feedback was sought. The training guide included links to reputable video clips and websites exploring how to deliver specific activities such as hand massages and range of motion exercises based on the suggestions of caregivers. The System for Classifying Modifications to Evidence-Based Programs or Interventions [44] was used to guide the adaptation process of Namaste Care, document changes made to the original Namaste Care program to assess fidelity, and as a coding framework for qualitative analysis. Key results of the adaptation process that informed the adapted Namaste Care program were that the program is expected to be integrated into daily care routines, tailored to individual preferences and abilities of persons with dementia, and delivered twice a week instead of twice a day as per the original program to fit the realities of caregiving. All of the activities included in the original Namaste Care program were left intact based on the recommendations of caregivers: (a) using a personalized approach; (b) integrating comfort and pain management; (c) scenting a room; (d) playing relaxing/energetic music; (e) providing touch activities; (f) offering beverages and snacks; (g) encouraging range of motion activities; and (h) providing reminiscence activities (e.g., photo albums, conversations about life stories) [45]. In the original Namaste Care program these activities can be further tailored based on preferences, cultural values, and life stories.

As part of this study, caregivers each received a Namaste Care Toolbox by mail at no cost. MY had conversations with caregivers about items to include prior to sending the Namaste Care Toolbox. Items were tailored to the preferences and abilities of each person with dementia. Items were also tailored to caregivers based on discussions regarding their comfort level in delivering activities and interests of care recipients and what items can be used as shared activities such as completing puzzles together or applying lotions for each other. Tailoring of items and activities was achieved through individual meetings with each caregiver scheduled one to two weeks prior to training to discuss the favorite activities and current abilities of persons with dementia, life stories of persons with dementia and caregivers, and what activities caregivers feel comfortable in delivering. The tailoring process was an important component of the adapted Namaste Care program to match activities to stages of dementia as persons with advanced dementia have been found to most often be provided with simple physical and sensory-based activities [46]. Ensuring that persons with dementia are provided with meaningful, favorite activities can increase the potential for positive benefits such as a decrease in depression and behavioural symptoms and an improvement in functional health [47]. Standard items included a training guide, Namaste Care activities checklist forms, a plastic storage container, an aromatherapy diffuser with lavender essential oil, hand and/or body lotion, a squeeze ball, a lip balm, and a microwavable magic bean hot and cold compress. In addition to standard items caregivers received items based on the personal interests of each person with dementia such as dolls, face cream, hairbrushes, fleece blankets, manicure sets, jigsaw puzzles, wordsearch puzzles, light-up balls, blankets, arts and crafts kits, painting supplies, granola bars, and/or pudding. Some caregivers also used items that they already had in their possession to deliver the adapted Namaste Care program sessions such as trivia games or gardening supplies. Participants received a 30 to 60-min virtual training session by phone or by Zoom on implementing the adapted Namaste Care program at home. Initial training content included: (1) examples of Namaste Care sessions; (2) key considerations and strategies for delivering and selecting Namaste Care activities (e.g., music, aromatherapy, massages, snacks and beverages, range of motion/exercises, reminiscence activities); (3) how to perform observational assessments of mood, alertness, and pain for persons with dementia before, during, and after Namaste Care; (4) examples of responses of persons with dementia to activities; and (5) strategies to optimize the delivery of Namaste Care (e.g., modifying complexity of activities, timing of activities, and making activities more engaging). MY provided bi-weekly check-ins over 3 months of program implementation with caregivers to assess their needs regarding additional training or material resources, address their questions, and provide information such as links to reputable video clips and websites. Additional information and training provided to caregivers following the initial training session included communication strategies, changes related to the progression of dementia, and finding activities that can be enjoyed by both persons with dementia and caregivers. Caregivers were provided with hands-on demonstrative video clips on how to perform a hand or foot massage and deliver gentle exercises or range of motion activities. Caregivers also communicated by email if they had any questions or concerns between check-ins.

## Aim and research questions

The aim of this mixed methods feasibility study was to determine the feasibility, acceptability and preliminary effectiveness of the adapted Namaste Care program. The primary aim of the study was to explore feasibility and acceptability of the adapted Namaste Care program, from the perspective of caregivers. The secondary aim was to determine preliminary effectiveness of the adapted program on selected caregiver outcomes. The primary research question was: What are caregivers’ views regarding the feasibility and acceptability of the adapted Namaste Care program? The secondary research question was: What are the preliminary effects of the program on caregiver QOL, perceptions of caregiving, self-efficacy and burden? The overarching mixed methods research question was: To what extent and in what ways do the findings from the qualitative interviews with caregivers contribute to a more comprehensive and nuanced understanding of the feasibility, acceptability and potential effectiveness of the adapted Namaste Care program delivered by caregivers in a home setting?

## Methods

### Multiphase mixed methods design

**Fig. 1:**
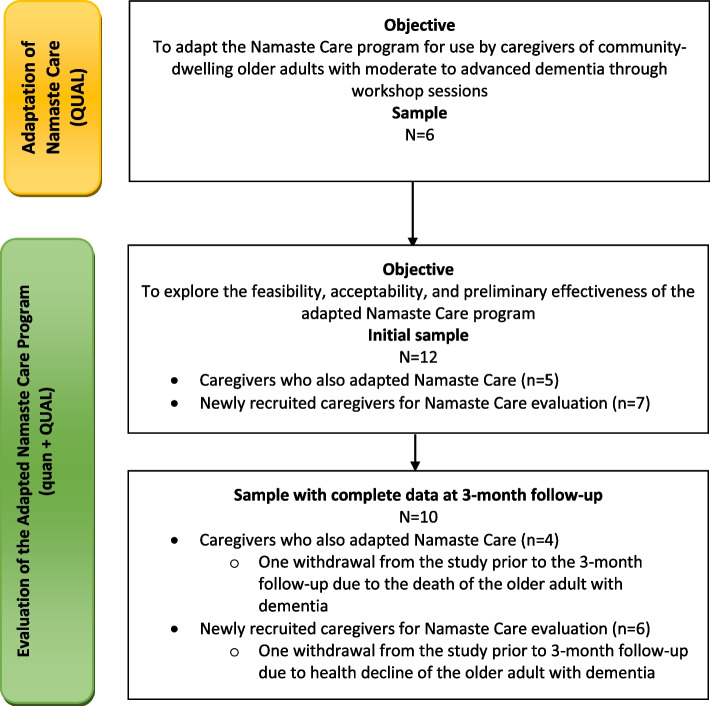
Mulitphase mixed methods study flow diagram

This feasibility study was part of a larger study using a multiphase mixed methods design which incorporated quantitative and qualitative approaches as well as sequential and concurrent strands over time to address an overall program objective [48]. The overall program objective was to adapt Namaste Care and evaluate its feasibility, acceptability, and preliminary effects for caregivers. The notation of the study design was QUAL —˃ [quan + QUAL] because we began the program of research using a qualitative strand to adapt Namaste Care, which is discussed elsewhere [43], and then evaluated the adapted program using quantitative and qualitative methods. The focus of this paper is on the [quan + QUAL] components. See Fig. 1 for study flow diagram of strands. The qualitative strands were given priority because the focus was on the feasibility of the study methods and procedures and to learn about caregivers’ perceptions of and experiences with delivering the Namaste Care program. In this study feasibility is defined as whether the adapted Namaste Care program can be used in a home setting and how should this be done. The aim was to understand feasibility and acceptability of the adapted Namaste Care program by allowing caregivers to share their experiences. The study protocol is published elsewhere [49].

#### Quantitative study design

A single group, before-after design was used to assess feasibility of the adapted Namaste Care program and assess preliminary effects for QOL, perceptions of caregiving, self-efficacy, and burden of caregivers. A before-after design, also called non-randomized design, was selected as it is often used in feasibility studies where the focus is on understanding the delivery process relating to the intervention arm, thus randomization is not essential [50]. The design is also cost-effective in assessing preliminary outcomes to determine whether further research is needed and eliminates the ethical challenge of assigning caregivers to a control group where they may not benefit from the intervention immediately [41, 51, 52]. The feasibility study is reported as per the guidelines of the Consolidated Standards of Reporting Trials (CONSORT) for pilot and feasibility studies [53]. Since the current study consists of a one-group, pre-post feasibility study, certain criteria of the CONSORT guidelines related to a randomized controlled trial do not apply.

#### Qualitative study design

Qualitative description was used to explore the acceptability of the program among caregivers, the experiences of caregivers in using the adapted Namaste care program, and to help explain preliminary effects of the program [54. ]. This design was selected to remain close to the words of the participants while giving room for some interpretation of data [54].

### Setting, participants and recruitment

#### Setting

Individual interviews took place virtually due to the COVID-19 public health restrictions at the time of the study. Individual training sessions for the adapted Namaste Care program occurred virtually using videoconferencing or by phone.

#### Participants

Types of purposive sampling used to select caregivers were criterion and snowball sampling [55]. Criterion sampling was used to seek participants who met the following study inclusion criteria: (a) aged 18 years or older; (b) currently providing physical, emotional, and/or psychological support in a home setting for at least four hours a week for a family member or friend with moderate to advanced dementia who is aged 60 years or older; (c) able to speak, write, and understand English; (d) currently living in Canada; and (e) able to provide informed consent. Four hours was selected to be included in the study criteria as it was reasonable to assume that the adapted Namaste Care program would require a minimum of fours hours per week for delivery. In this study caregivers were defined as unpaid caregivers who provide support or physical care for a family member or friend. Caregivers did not have to assume the role for a certain amount of time to be eligible for the study and did not have to live with persons with dementia. For example, adult children caregivers may not be living with their parents but still provide at least four hours a week of care for a parent with dementia. Stages of dementia were determined using the Reisberg/Global deterioration scale based on what was reported by caregivers [56].

Snowball sampling was also used by asking caregivers to share study and researcher contact information with other caregivers who may be interested in participating in the study [55]. A total of 10–20 caregivers was sought and 12 were recruited. In feasibility studies, a primary outcome and power calculation for sample sizes may not be necessary [57]. The sample size for caregivers was determined based on previous Namaste Care studies which included anywhere from 8 to 15 caregivers [32, 35, 58–61]. Some of the previous Namaste Care studies reporting the inclusion of a small number of caregivers consisted of a feasibility study and a feasibility study using a mixed methods design [58, 59]. Convenience sampling [55] was also used as caregivers who participated in adapting Namaste Care were interested in delivering the program and receiving supports and were therefore invited to participate in the evaluation phase of the study. We recognize that these caregivers would be quite motivated to help with the study but felt it was not ethical to withhold this intervention from these participants. Computer tablets and access to the internet were offered to caregivers who did not have access to these and who were interested in participating by videoconferencing.

#### Recruitment

Caregivers were recruited from the Alzheimer Society of Canada, local and provincial Alzheimer Societies, Dementia Advocacy Canada, provincial and local caregiver organizations, and community-based geriatric services. MY presented the study virtually for caregivers and Alzheimer Society staff (e.g., public education coordinators, counsellors, recreational therapists). MY presented the study at virtual Alzheimer Society dementia education series for caregivers (e.g., Care in the Later Stages Series). Information about the study was shared through websites, social media, and in electronic newsletters of the listed organizations.

### Data collection and analysis

Data were collected from October 2020 to September 2021. Demographic data, such as age, sex, education level, relationship to care recipient, ethnicity, and chronic conditions, were collected using questionnaires and analyzed using descriptive statistics. Means and standard deviations were reported for continuous variables and frequency counts and percentages were reported for categorical variables. Baseline data including demographic and outcome data (i.e., QOL, perceptions of caregiving, self-efficacy, and burden) were collected immediately after informed consent was obtained. A research activity log was completed bi-weekly to document the number of days the adapted Namaste Care program was used per week, specific activities conducted during the sessions, and the number and type of adverse events reported. At the end of the 3-month intervention period, outcome data were collected again along with qualitative data (e.g., acceptability, perceived benefits, barriers and facilitators to implementation). Post-intervention data were collected within two-weeks following the end of the intervention period. Quantitative data analyses were conducted using SPSS version 28. The statistical software used for multiple imputation was the MICE package in base R Version 4.0.2 [62]. All tests were completed using 95% confidence intervals (CI) and a two-sided alpha level set at 0.05.

#### Feasibility

Data on feasibility were collected by completing the research activity log. MY updated the log based on information obtained from caregivers (e.g., Namaste Care Activities Checklist and notes) during the bi-weekly telephone or videoconference check-ins. Caregivers were provided with a Namaste Care Activities Checklist that listed common activities that can be delivered during a session. Descriptive statistics were used to summarize feasibility data obtained through the research activity log and Namaste Care Activities Checklists completed by each caregiver to provide an overview of different types of Namaste Care activities delivered by caregivers and the frequency of activities delivered. Caregivers were asked to complete a brief Namaste Care Activities Checklist on the days they delivered the program to indicate the type of activity delivered during a session such as massage, aromatherapy, snacks, beverages, and music [See Additional file 1 for the checklist]. The Namaste Care Activities checklist was also used to assess fidelity by ensuring that caregivers delivered the Namaste Care activities listed on the checklist. Fidelity of the intervention was established by implementing standardized training protocols and analyzing the Namaste Care Activities checklist to measure adherence to the intervention protocol [63]. Feasibility was evaluated by comparing the research activity log to pre-set target criteria: (a) number of days the adapted Namaste Care program was used (target: at least 2 out of 7 days per week for at least 8 out of 12 weeks for participants who delivered the program over 3 months); (b) retention rate (target: 75% of participants have completed the 3-month outcome data questionnaires and interviews); and (c) percentage of adverse events for persons with dementia (e.g., falls, injuries, skin breakdown) reported (target: 0%). If a caregiver combined two or more activities in a session, the adapted Namaste Care program was considered as being implemented on that day. The target for the number of days the Namaste Care program was used was based on the feedback provided by caregivers when adapting the program and used in another study evaluating Namaste Care in LTC [58]. Results were also reported in relation to whether pre-set target criteria of the study were met.

#### Acceptability

The acceptability of the adapted Namaste Care program was explored through individual interviews with caregivers by phone or by videoconference at the end of the study period (i.e., at 3-month follow-up). These interviews explored caregiver perceptions of the following: (a) benefits of the program; (b) satisfaction with the process of implementation; (c) barriers and facilitators to implementation; and (d) recommendations to improve the program. Qualitative data were collected bi-weekly through notes and at the 3-month follow-up which consisted of a 30- to 60-min individual virtual (i.e., telephone or videoconference) semi-structured interview with caregivers to explore their experiences in implementing the adapted program. The interview guide was used to explore experiences with the program and acceptability. See Table 1 for the interview guide. The aims of the qualitative strand and the interview guide were developed based on the Bowen et al. (2009) Feasibility Framework [39], semi-structured qualitative research methods [64], recommendations by research team members and dementia literature. Reflective field notes taken immediately after the interviews were completed to capture actions and to highlight personal reactions by bringing important elements to light such as emotions experienced by participants [65]. Interviews were audio-recorded and transcribed by an experienced transcriptionist.

**Table 1:** Caregiver post-intervention interview guide

Delivering the Adapted Namaste Care Program:
1. What were your experiences in delivering the program at home? 2. How did you feel about the resources provided to you to use the program at home? 3. How confident and comfortable did you feel in using the program at home? 4. How supported did you feel in implementing the program at home? 5. How often and over what length of time were you able to deliver the program? 6. What kind of activities did you provide for the person you are caring for? 7. How has the program changed the way you deliver activities? 8. What made it difficult to use the program? 9. What made it easy to use the program? 10. How did you personalize care activities for your family member or friend?
Satisfaction with the Adapted Namaste Care Program:
11. Would you continue to use the program at home after this study is done? Why or why not? 12. How satisfied were you in using the program? 13. What changes would you recommend to improve the program?
Effects of the Adapted Namaste Care Program:
14. What impact did Namaste Care have for you? 15. What impact did Namaste Care have for your family member or friend? 16. Has Namaste Care created any changes in how you see your role as a caregiver? If so, what kind of changes? 17. What impact did Namaste Care have on your connection and relationship with your family member or friend? 18. Did you feel like delivering Namaste Care made a difference for you and/or your family member or friend? In what ways?

Some of the questions included in the guide were inspired by the Bowen Feasibility Framework (Bowen et al., 2009) [39]

Qualitative data obtained from the interviews were analyzed using experiential thematic analysis, which is suitable for the study as it focuses on the experiences of participants and how they make sense of their world [66, 67]. Analysis was both inductive and deductive in nature. Categories for qualitative themes were developed based on selected areas of focus of the Bowen et al. (2009) Feasibility Framework [39] including acceptability, demand, implementation, practicality, efficacy, and adaptation. The analytical process followed the six phases of Braun and Clarke’s (2006) thematic analysis [66]: (a) gaining familiarity with the data; (b) conducting coding; (c) locating themes; (d) reviewing themes, (e) developing a definition for themes and naming them; and (f) developing a report. MY analyzed data concurrently as interviews were being completed and then shared findings with the research team for feedback. Qualitative description allows researchers to begin analysis with the use of pre-existing coding systems (i.e., Bowen Feasibility Framework) [39] and adapting these systems during the analysis to ensure they fit the data [54]. Consistent with qualitative description [54], constant comparative analysis was used to identify similarities and differences across participants. NVivo version 12 software [68] was used for data management.

#### Preliminary effectiveness

Preliminary effectiveness data were collected at baseline and at the 3-month follow-up. Preliminary effectiveness was assessed using four caregiver outcomes – QOL, perceptions of caregiving, self-efficacy, and burden. QOL was measured using two subdomains from the Carers-DEMentia Quality of Life (C-DEMQOL) scale for obtaining the QOL of caregivers of people with dementia [69]. To alleviate the burden for caregivers in completing a 30-item questionnaire, relevant subdomains of the scale were used in the study (i.e., carer wellbeing and carer role). These subdomains were selected as they closely resembled goals of the Namaste Care program such as enhancing the relationships between persons with dementia and caregivers [28]. The other subdomains were excluded because they focus on perceptions of the future and social and professional support, which were not a focus of the Namaste Care program. Other effectiveness measures included caregivers’: (a) perceptions of caregiving measured using the 9-item Positive Aspects of Caregiving (PAC) scale [70]; (b) self-efficacy measured using the Relational, Instrumental, Self-soothing (RIS) Eldercare Self-Efficacy scale [71]; and (c) caregiver burden measured using the short form Zarit Burden Interview scale (ZBI-12) [72]. Caregivers were provided with the option to complete questionnaires independently or with MY by phone or videoconference.

The instruments were carefully selected based on validity and reliability and whether they were specific for caregivers of people with dementia and fit the home setting context. For the two C-DEMQOL subdomains that were used in the study, each was composed of six items, carer wellbeing had a Cronbach alpha of 0.91 and care-patient relationship/carer role had a Cronbach alpha 0.82; scores range from 6–30 for each of these two subdomains, and higher scores reflect better QOL [69]. For the PAC nine-item measure the Cronbach alpha for the scale was 0.89; scores range from 9–45, and higher scores reflect greater positive feelings towards caregiving [70].

The RIS Eldercare Self-Efficacy scale is a 10-item tool that measures different forms of self-efficacy or perceived belief in being able to complete an activity [70]. Instrumental self-efficacy consists of one’s belief in being able to complete personal care tasks for the care recipient; relational self-efficacy consists of one’s belief in maintaining a positive relationship with the care recipient; and self-soothing efficacy is one’s belief in being able to maintain one’s own wellbeing amidst all the demands of caregiving [70]. Scores range from 10–50 with higher scores reflecting greater self-efficacy. Internal consistency was established for relational self-efficacy with a Cronbach alpha of 0.73, 0.78 for instrumental self-efficacy and 0.72 for self-soothing self-efficacy [70]. The validity of the instrument was established with the ability to explain 61.2% of total variance using a principal component factor analysis [70].

The ZBI-12 [72] measures caregiver burden and is a short (12-item) version adapted from the 22-item and the original 29-item scales created by Zarit et al. (1980) [73]. Scores range from 0 to 48 with higher values representing greater burden. Internal consistency was established with a Cronbach alpha value of 0.88 found for the ZBI-12 scale [72].

Normalcy was assessed using Shapiro–Wilk tests to determine whether the data were normally distributed before deciding to conduct parametric or non-parametric tests to assess the statistical significance of the change in scores from baseline to 3 months for the measures of effectiveness. Based on the findings paired t-tests were used. A secondary analysis explored the impact of missing data (18% missing for the 3-month outcomes), with multiple imputation employed using chained equations, predictive mean matching and 5 imputed data sets [74]. Multiple imputation was considered appropriate because the proportion of missing data exceeded 5%, a common rule of thumb for deciding not to ignore missing data. The proportion of missing data was also not so large (e.g., above 40%), that using the existing data for imputation would be misleading [74]. We further assumed the data were missing at random (MAR), which is the starting point for most modern missing data analyses because MAR is more general and realistic compared to alternative missingness patterns [75]. MAR also seemed consistent with the scenarios applicable to the two caregiver dropouts (which were related to the death and health decline of the person with dementia). As this study targets older adults with moderate to advanced dementia who are progressing towards the end of life, it is inevitable that death or health decline might occur in this population during the study, potentially resulting in caregiver dropouts and missing data. Missing data are very common in palliative and end-of-life studies where 20–50% of participants drop out due to health decline or death [76]. The events that lead to dropouts are often unpredictable making it difficult to exclude the individuals. Exclusion is undesirable in any case because the study results are less likely to be representative of what would be seen in practice. Therefore, caregiver dropouts were kept in as a secondary analysis and multiple imputation was used to address missing data. Since the missing data pattern was uncertain, a MAR pattern was assumed consistent with most modern missing data methods [75]. Due to the small sample size, the multiple imputation analyses were regarded as exploratory [74]. Pooling was done using mi.t.test routine in MKmisc package. All variables (e.g., demographic, health status, caregiver outcomes) were included in the imputation model. The 5 imputations were pooled to estimate the treatment effect for each caregiver outcome.

#### Mixed methods analysis

Once data analysis for both the quantitative and qualitative strands were completed, the findings of both analyses were compared to develop the mixed methods interpretation [48]. Meta-inferences were made at the end of the study by combining the inferences from quantitative and qualitative strands to provide a broader interpretation [48, 77]. A joint display was used to show the connection between quantitative results and qualitative findings that either supported or diverged from one another [48]. The Bowen et al. (2009) Feasibility Framework was used to develop the mixed methods interpretation and inform the joint display structure that included feasibility, acceptability, and preliminary effectiveness data [39].

### Ethical considerations

Ethics approval was received from the Hamilton Integrated Research Ethics Board (#10,526). All methods were carried out in accordance with the Tri-Council Policy Statement: Ethical Conduct for Research Involving Humans which includes three core principles, respect for persons, concerns for welfare, and justice [78]. All caregivers provided verbal informed consent approved by the Hamilton Integrated Research Ethics Board, and this was audio-recorded. Caregivers were afterwards provided with a signed copy of the consent form. A token of appreciation in the form of a $25 gift card was offered to all caregivers for participating in the study.

### Validity, rigour, and trustworthiness

#### Quantitative validity

When selecting the quantitative instruments, we considered content validity to ensure that the instruments measured relevant aspects of caregiver outcomes and face validity to ensure that the measurement and number of items included appeared reasonable [78]. The instruments selected were relevant to the target population of the study and were used in previous studies that evaluated effectiveness of caregiver interventions [26, 80–85]. They were also selected based on the outcomes anticipated to be impacted by the adapted Namaste Care program.

#### Qualitative validity

MY maintained a reflexive journal to document reactions and experiences potentially having an impact on the study process [86]. We implemented strategies to meet Lincoln and Guba’s (1985) trustworthiness criteria consisting of credibility, transferability, dependability and confirmability [87]. Credibility was upheld through investigator triangulation by seeking feedback from research team members as they hold research expertise in the areas of caregiver support, dementia care, psychosocial interventions and/or community support programs. This process promoted credibility and complementarity as well as validated data [87]. To increase the transferability of the study findings, rich thick descriptions were used to describe the study setting and sample [87]. MY conducted a study audit to meet the criteria of dependability and confirmability to ensure that the process for collecting and analyzing data was systematic and findings were supported by data.

#### Mixed methods study validity

We implemented strategies to minimize threats related to data collection, data analysis and interpretation of both quantitative and qualitative findings. We used data collection methods that were appropriate for quantitative and qualitative methods [48]. We upheld validity by integrating quantitative and qualitative strands to answer the mixed methods research question and revealing convergences and divergencies between the findings of both strands in a joint display. The Mixed Methods Appraisal Tool (MMAT) was used to ensure that methodological quality criteria unique to mixed methods studies were considered in the current study such as adequate justifications for selecting a mixed methods design, effective integration of qualitative and quantitative strands, and clear reporting of divergences and consistencies between qualitative and quantitative data [88].

## Results

In this section we present quantitative and qualitive findings and mixed methods interpretations. We begin by providing demographic information of participants followed by feasibility, acceptability, and preliminary effectiveness findings. We summarize the overall findings in the section on mixed methods interpretations.

### Demographics characteristics

A total of 12 caregivers participated in the study and implemented the adapted Namaste Care program at home, however 10 caregivers completed the study. The reasons for study withdrawal were due to the death of the spouse with dementia and a parent with dementia experiencing a decline in health status. The demographic data of the 12 participants who enrolled in the study are discussed here. The mean age of caregivers was 62.3 years [SD (standard deviation = 11.8)]. Most of the caregivers were female (83.3%). Caregivers were from four Canadian provinces with most living in Ontario (66.7%) and in urban areas (83.3%). All except one caregiver identified as being White/Caucasian (91.7%). The one caregiver identified as being Chinese. A little over half of caregivers were retired from paid work (58.3%) and most had completed post-secondary education (91.6%). There was an equal number of caregivers who were children versus spouses of persons with dementia (50%). The mean number of years since caregivers took on the caregiving role was 5.1 years [SD = 3.6]. In terms of number of chronic conditions most caregivers reported anywhere from 1 to 4 conditions (83.3%) such as thyroid disorders, chronic musculoskeletal conditions causing pain or limitations, hypertension, and gastroesophageal reflux disease. Most caregivers (75%) were responsible for providing all types of support such as emotional support, assistance with household activities, and assistance with personal care for persons with dementia. See Table 2 for the demographic characteristics of caregivers.

**Table 2:** Demographic characteristics of caregivers (*N* = 12)

Category	n (%)
Age in years (Mean [SD])	62.3 [11.8]
30–49	1 (8.3)
50–59	4 (33.3)
60–69	4 (33.3)
70 and older	3 (25)
Sex	
Female	10 (83.3)
Male	2 (16.7)
Province of residency	
Ontario	8 (66.7)
Manitoba	2 (16.7)
Alberta	1 (8.3)
British Columbia	1 (8.3)
Geographical area of residency	
Rural	2 (16.7)
Urban	10 (83.3)
Ethnicity	
White/Caucasian	11 (91.7)
Chinese	1 (8.3)
Highest level of education completed	
High school diploma	1 (8.3)
College diploma	3 (25)
Bachelor’s degree	3 (25)
Graduate or professional degree	4 (33.3)
Other: Post secondary course	1 (8.3)
Employment status	
Working full-time	3 (25)
Working part-time	2 (16.7)
Retired from paid work	7 (58.3)
Relationship to person with dementia	
Son/daughter	6 (50)
Spouse	6 (50)
Number of years as a caregiver (Mean [SD])	5.1 [3.6]
2–4	6 (50)
5–7	5 (41.7)
8 and up	1 (8.3)
Number of chronic conditions	
None	2 (16.7)
1–2	7 (58.3)
3–4	3 (25)
Type of support provided	
Advice or emotional support and assistance with household tasks	3 (25)
Advice or emotional support, assistance with household tasks, and assistance with personal care	9 (75)

The total value of some categories does not equal 100% due to rounding

In terms of the demographic characteristics of persons living with dementia, which were reported by caregivers, the mean age was 76.9 years [SD = 9.5]. A little over half of persons with dementia were male (58.3%). The mean number of years that an individual was diagnosed with dementia was 4.8 [SD = 4]. Most persons with dementia were in the moderate stages of dementia (66.7%) versus the advanced stage (33.3%). Using the Reisberg/Global deterioration scale, persons with dementia at stages 5 (moderate) or 6 (moderately severe) were considered as having moderate dementia and those at stage 7 (severe) consisting of the last stage of the scale were considered as having advanced dementia [56]. Persons with dementia had many chronic conditions with most having 3 to 6 conditions (91.7%), such as depression or anxiety, cardiovascular disease, hypertension, hyperlipidemia, and chronic musculoskeletal conditions causing pain or limitations. See Table 3 for the demographic characteristics of persons living with dementia.

**Table 3:** Demographic characteristics of persons living with dementia (*N* = 12)

Category	n (%)
Age in years (M [SD])	76.9 [9.5]
60–69	3 (25)
70–79	3 (25)
80 and older	6 (50)
Sex	
Female	5 (41.7)
Male	7 (58.3)
Number of years diagnosed with dementia (Mean [SD])	4.8 [4]
1–3	4 (33.3)
4–6	5 (41.7)
7 and up	3 (25)
Stage of dementia	
Moderate	8 (66.7)
Advanced	4 (33.3)
Number of chronic conditions	
1–2	1 (8.3)
3–4	6 (50)
5–6	5 (41.7)

The total value for some categories does not equal 100% due to rounding

### Feasibility results

We present results that provide evidence for the feasibility in implementing the adapted Namaste Care program and the types of activities that were feasible to deliver by caregivers. All 10 caregivers who completed the study met the pre-set target criterion of using the adapted Namaste Care program for a minimum of twice per week for at least 8 out of the 12 weeks of the study. Eighty-three percent of the caregivers (10 of 12) completed the study, which exceeded the target retention rate of 75% or more. No adverse events related to program delivery were reported by caregivers.

#### Type and frequency of activities delivered

Caregivers were asked to document activities delivered during sessions using the Namaste Activities checklist [See Additional file 1]. All caregivers combined at least 2 activities into a session. Providing snacks and beverages outside of mealtimes was the most frequently completed activity with 83.3% of caregivers taking part in this activity and doing so at least 5 times a week on average. In terms of reminiscing activities (e.g., storytelling, looking at photo albums or family videos), 75% of caregivers delivered this activity weekly for 4 days a week on average. Physical touch activities (e.g., washing and/or moisturizing hands, face and/or feet, massaging) were provided by 66.7% of caregivers and delivered 3 times a week on average. Range of motion/exercise-based activities (e.g., walking, exercise routines, playing with a ball) were delivered by 66.7% of caregivers and offered 3 times a week on average. Audio/visual activities (e.g., reading, listening to music, watching video clips) were provided by 66.7% of caregivers twice a week on average.

Comforting activities (e.g., providing a hot/cold pad, implementing aromatherapy, offering a soft blanket) were delivered by 58.3% of caregivers for 3 times a week on average. Despite COVID-19 public health restrictions, caregivers still found alternative ways to provide opportunities for socialization for persons with dementia. Family/friend visits (e.g., phone or Zoom calls, in-person visits when safe to do so) and outings were provided by 58.3% of caregivers at least twice a week on average. Games (25%) and arts and crafts (0.8%) were the least frequently delivered activity with both being delivered once a week on average. See Table 4 for the type and frequency of activities delivered during program sessions.

**Table 4:** Type and frequency of activities delivered by caregivers during the adapted Namaste Care program sessions

Type of Activity	Average number and percentage of caregivers that delivered the activity weekly	Average number of days the activity was delivered per week
**Snacks and Beverages** -Flavored soda water -Coffee or tea -Milkshakes or smoothies -Dessert -Fruits -Pudding -Yogurt -Jell-O -Granola bars	10 (83.3%)	5
**Reminiscing** -Telling stories -Talking about family letters -Looking at photo albums or family videos	9 (75%)	4
**Physical Touch** -Face, hands, and/or feet washed and/or moisturized -Applied lip balm -Fingernails cleaned and/or clipped -Hair brushed or combed -Massage (Head/face, feet/legs, hand/arms, back)	8 (66.7%)	3
**Audio/visual** -Reading newspapers or magazines -Watching movies or shows -Listening to music -Watching video clips -Watching live virtual concerts	8 (66.7%)	2
**Range of Motion/Exercise** -Exercise videos -Walking outdoors -Walking up and down the stairs -Dancing -Yoga -Playing with a ball -Water workouts -Golf -Bike -Household chores (Dishes, yard work)	8 (66.7%)	3
**Family/friend visit and/or outing** -Phone or Zoom Calls -In-person visit -Outing to farmer’s markets, greenhouses, beach, park, etc	7 (58.3%)	2
**Comforting** -Aromatherapy (Electronic diffuser (lavender), flowers) -Hot/cold Pad -Fleece blanket -Meditation -Holding hands	7 (58.3%)	3
**Games** -Jigsaw puzzles -Wordsearch -Trivia -Checkers -Card games (Go Fish, Old maid)	3 (25%)	1
**Arts and Crafts** -Baking -Collages -Gardening -Play-Doh	1 (0.8%)	1

### Acceptability findings

Qualitative themes were categorized under the following: (a) program implementation experiences of caregivers; (b) facilitators supporting program delivery; (c) barriers to program delivery; (d) perceived benefits for caregivers; (e) perceived benefits for persons living with dementia; and (f) recommendations for further adaptations. See Table 5 for an overview of categories and themes.

**Table 5:** Overview of qualitative themes

Category	Theme
Program Implementation Experiences of Caregivers	Caregivers were highly satisfied with the program resources and training
	Delivering the program was manageable and fit into caregivers’ routines
	Bi-weekly check-ins helped caregivers deliver the program confidently and consistently
Facilitators Supporting Program Delivery	Being provided with a Namaste Care Toolbox
	Having written resources at hand
	Delivering activities in a language familiar to persons living with dementia
Barriers to Program Delivery	Persons living with dementia did not want to engage in activities at times
	Persons living with dementia did not respond to all items provided in the Namaste Care Toolbox
	Meeting the numerous daily demands (e.g., work, housework, appointments)
Perceived Benefits for Caregivers	The program encouraged a more structured approach and use of creativity in delivering activities
	Caregivers gained a better understanding of how to address the various needs (e.g., physical, emotional, social) of persons living with dementia
	The program brought caregivers and persons living with dementia closer in their relationships through mutual enjoyment of activities
Perceived Benefits for Persons Living with Dementia	Persons with dementia were more engaged in meaningful activities consistently
	Enhanced wellbeing of persons living with dementia
	The program instilled confidence in persons living with moderate dementia to participate in different activities
Recommendations for Further Adaptations	Offering more caregiver training and information on how to involve others (e.g., other family members, personal support workers) in delivering the program
	Further tailoring of items provided in the Namaste Care Toolbox to increase the likelihood of engagement
	Reformatting the Namaste Care activities checklist

#### Program implementation experiences of caregivers

Caregivers reported positive experiences in implementing the adapted Namaste Care program at home. Themes were: (a) caregivers were highly satisfied with the program resources and training; (b) delivering the program was manageable and fit into caregivers’ routines; and (c) bi-weekly check-ins helped caregivers deliver the program confidently and consistently.

##### Caregivers were highly satisfied with the program resources and training

All caregivers reported high levels of satisfaction in delivering the adapted Namaste Care program and stated that they would continue using the program after the study was completed. “*There are tools in my toolbox as the result of Namaste Care that I will take forward*” (CG-211). They found value in using the program as it gave them various ideas for activities to engage older persons with dementia. Some caregivers planned to try different activities with their family members (e.g., outdoor activities, painting sessions) and one caregiver planned to complete a chart to track activities. Caregivers perceived that this program would be helpful for other caregivers, and many shared the program with other caregivers and family members. “*Extremely satisfied [with the program]. I think it would be great for everybody to have that opportunity, to have that help, that program, that support, all of it. I think it’s fantastic”* (CG-204). Caregivers were also satisfied with the training session, resources, and items included in the Namaste Care Toolbox. “*[When asked about training] Excellent. The whole idea of how to incorporate things. It was all so helpful. Like any input is like gathering information and then using what you can of it in your situation”* (CG-212).

##### Delivering the program was manageable and fit into caregivers’ routines

Caregivers perceived that delivering the program at least twice a week was manageable in terms of meeting other priorities such as work and daily household tasks. Some caregivers were even able to deliver the program daily. The program did not create additional burden for caregivers and provided them with a different approach to care. “*It didn’t feel like I was doing any extra work, it just sort of changed the pace of things that I would do for the caregiving”* (CG-205). The program was perceived as complementing the routines of caregivers. They reported providing some activities that were on the checklist before, but the program helped them in *“making it [activities] a little bit more special”* (CG-212). They also felt comfortable in delivering the program sessions because some activities were familiar to them.

##### Bi-weekly check-ins helped caregivers deliver the program confidently and consistently

Caregivers reported that having regular bi-weekly meetings with MY were helpful to provide them with feedback regarding program delivery, answer questions, and support them to try different ideas for activities. The consistent support provided confidence and encouragement for caregivers to continue with the delivery of the program. *“It’s good just to have that social feedback and… every two-weeks is about right. And it enabled me to sort of stay focused as well”* (CG-203). Caregivers appreciated receiving validation for their efforts in delivering the program and appreciated receiving website links to help address their needs.

You reassuring me that I am actually doing quite a lot when sometimes I feel like I am not, you know or where I intended to spend longer, I think it helped me feel better, so that was very valuable. And sometimes I tell you the struggles that we had, just as it comes. Not, thinking that you had any suggestions, but then you would send me some resources, or you will send me some links. That was useful for sure.(CG-206)

#### Facilitators supporting program delivery

Caregivers perceived that they were well supported to deliver the adapted Namaste Care program in terms of training and education, material resources, and regular communications. They were also comfortable in ensuring the program was tailored to the preferences, needs, and abilities of persons with dementia. Themes for program delivery facilitators were: (a) being provided with a Namaste Care Toolbox; (b) having written resources at hand; and (c) delivering activities in a language familiar to persons living with dementia.

##### Being provided with a Namaste Care Toolbox

Caregivers perceived that being provided with a Namaste Care Toolbox at no cost made it easy to implement the program because caregivers may not have the time or expertise or financial resources to purchase items on their own. *“If you had to go out and purchase them yourself, the uncertainty, ‘did I get the right thing?’ It was super easy, I opened the box and utilized it. So, there wasn’t any guess work”* (CG-211). When caregivers wanted to deliver a session, they did not need to search for items as all items were located in one container.

Well, it was nice to have all the stuff there all in the box. I kept them in the box and container. I appreciated you bringing them and having them all there. And you know, when I was done, I just put it back so that everything is all there. So, that helped. (CG-204)

##### Having written resources at hand

Caregivers were provided with a training manual and additional resources (e.g., links to videos or websites, research articles) that were tailored to their learning needs. Having written resources available supported program delivery so that caregivers could refer to information about the program as needed. They were given a checklist to record the activities provided and many referred to the checklist for ideas for activities.

It is great to have everything, you made it very easy because everything [resources] was all there if you didn’t give me the checklist, I have to record everything in my diary and think of things to write probably, but not too much information or detail. (CG-205)

##### Delivering activities in a language familiar to persons living with dementia

The adapted Namaste Care program is intended to be further tailored based on individual preferences, cultural values, and life stories. Caregivers perceived that this aspect of the program was important to consider when delivering the program for older persons with dementia to ensure they can relate and meaningfully engage in activities. All caregivers were expected to take part in the study in English, however some caregivers were supporting family members who no longer communicated in English and used their primary language when engaging in daily activities. Being able to play music or provide reading materials in one’s primary language made it easier for caregivers to engage with their family members.

Because for the music and the video I had to do things…that meant something to her. So, if I had to play something that I relate to, she may not relate to. She may not relate to it at all and not the language part. She can’t understand it. (CG-205)

#### Barriers to program delivery

Caregivers perceived that they experienced some challenges in delivering the adapted Namaste Care program. These were: (a) persons living with dementia did not want to engage in activities at times; (b) persons living with dementia did not respond to all items provided in the Namaste Care Toolbox; and (c) meeting the numerous daily demands (e.g., work, housework, appointments).

##### Persons living with dementia did not want to engage in activities at times

At times it was frustrating and/or disappointing for caregivers when they wanted to engage their family members in activities and felt they were not successful in doing so. Persons living with dementia were perceived as not being alert enough, not liking specific activities, and being too restless at times to participate in activities. Some persons with dementia did not want to engage in activities because they wanted to spend time alone.

The only thing is, if he wants alone time, more. Even…was it a couple nights ago? I went to go in to just chat with him or something. What did he say to me? You know, ‘what are you doing in here? Or ‘what do you want’… So, you know I find the fluctuation as time is going on*.* (CG-212)

##### Persons living with dementia did not respond to all items provided in the Namaste Care Toolbox

Despite tailoring items provided in the Toolbox as best as possible to the stages and individual preferences of persons with dementia, some individuals did not respond to all items in the Toolbox such as baby dolls. Caregivers were expecting that a response would be seen for most of the items such as items capturing the attention of persons with dementia or being held in their hands for longer periods. Caregivers perceived that when persons with dementia were in advanced stages there were greater limitations in activities that could promote a response such as a smile, eye contact, or attempts at verbal communication.

As in regards to any of the equipment and stuff because you provided a box of things, again unfortunately, [wife] appears to be beyond most of those. She wasn’t interested in sitting with a doll. Really, the squeeze ball and the one that lights up, she pays attention to that, if you bounce it around. The squeeze ball would be the one she spent the most time with and got the most attention*.* (CG-203)

##### Meeting the numerous daily demands (e.g., work, housework, appointments)

Most of the caregivers were primary caregivers who were living with their family member with dementia. They had numerous responsibilities in terms of work, household tasks, and managing appointments. Caregivers perceived that some activities proposed in the program conflicted with the time reserved to meet other responsibilities.

So, some of the things were specific, if you had time to say, ‘well, I wonder what I’ll do this morning. Oh, let’s get out the photo albums’, but in our household, there is so much that I need to be doing that I really don’t have the time. [husband] sleeps ‘till late, so I try to use that time to have a bit of time for myself, walk the dog, plan the day and you know, so it’s a bit complicated. (CG-208)

Some caregivers initially viewed the program as adding on to an already busy routine, but soon realized that the program could be integrated in everyday routines*. “It did become kind of common place after the first little while”* (CG-210).

#### Perceived benefits for caregivers

The adapted Namaste Care program was perceived by caregivers as having positive benefits for them. Themes were the following: (a) the program encouraged a more structured approach and use of creativity in delivering activities; (b) caregivers gained a better understanding of how to address the various needs (e.g., physical, emotional, social) of persons living with dementia; and (c) the program brought caregivers and persons living with dementia closer in their relationships through mutual enjoyment of activities.

##### The program encouraged a more structured approach and use of creativity in delivering activities

Caregivers perceived that the program provided them with a structured approach to deliver activities consistently and increased their efforts in forming connections with persons with dementia. *“More conscious in trying to take a structured approach… There is an effort to do something with her on daily basis which she can and hopefully responds too. And it’s worth it because, you get the great big smile…”* (CG-203). The program helped them to use creativity in delivering activities and awaken the different senses of persons with dementia.

I draw her attention to the different flavors that she is drinking. We often have like carbonated water, but it’s got a flavor in it. …the Play-Doh and that also felt really good for her…the Namaste got me expanded to think beyond just what sort of colouring can we do. (CG-206)

##### Caregivers gained a better understanding of how to address the various needs (e.g., physical, emotional, social) of persons living with dementia

Implementing the adapted Namaste Care program helped caregivers better understand the various types of needs persons living with dementia have and different strategies that they could use to address these. Caregivers perceived that prior to the program they were providing basic care needs for persons with dementia such as nutrition and now felt more confident in meeting other needs such as social and emotional needs. Some caregivers reported that they now recognized the needs of their family members amidst the overwhelming daily tasks.

It’s very easy and it’s really hard not to get caught up with the mental load with everything that has to get done in a day. And I think sometimes those tasks and chores are overloading. They are things that just have to get done, but this way you more see it as the person that you are looking after more of a person with needs, like we all have. (CG-202)

##### The Program brought caregivers and persons living with dementia closer in their relationships through mutual enjoyment of activities

Caregivers perceived that the program changed their relationship with their family members by bringing them closer and created more opportunities for interactions. They felt that the program helped them be patient and present when interacting with persons with dementia. Caregivers enjoyed taking part in activities and felt that these benefitted them as well. Some caregivers reported that persons with dementia would do activities for them such as applying lotion or providing a massage, and this provided opportunities for persons with dementia to lead activities. “*I found that [spouse applying lotion to my feet] would help to bring us together again, to have that touch”* (CG-209).

#### Perceived benefits for persons living with dementia

Caregivers identified many benefits of the program for persons living with dementia. Perceived benefits for persons with dementia were: (a) persons with dementia were more engaged in meaningful activities consistently; (b) enhanced wellbeing of persons living with dementia; and (c) the program instilled confidence in persons living with moderate dementia to participate in different activities.

##### Persons with dementia were more engaged in meaningful activities consistently

Caregivers perceived that the program ensured that persons living with dementia were provided with more frequent and regular opportunities to be engaged in meaningful activities. Persons with dementia were receiving much needed attention from caregivers and enjoyed pleasant conversations that did not require recall of events. Without this engagement caregivers perceived that family members would sleep or watch television.

I think she feels when we have a session like that she is being paid attention to and that she is doing something worthwhile because when I am not doing a session with her she is either sleeping or watching TV. So, doing something a bit more constructive, feels good for her because she has something to do and she feels more engaged. (CG-202)

##### Enhanced wellbeing of persons living with dementia

The program was perceived by caregivers as improving the wellbeing of persons with dementia through better mood following the delivery of pleasant activities and decreasing agitation. Aromatherapy and physical touch activities such as massages helped persons with dementia to relax.*“He is a little bit more relaxed and more engaged. And…it almost soothes him”* (CG-204). Caregivers reported positive responses such as smiling and increased verbal communication. One caregiver noted that his mother looked forward to the program every day and disruptions to its delivery had an impact on her.

It’s sort of a routine that she knew everyday and if that’s in the morning, the snacks, the beverage and the lotioning. So, I think the routine actually helped her get more settled. She knows she can look forward to that every day. And there was a time when I had to go out for meetings or had to go out for groceries, the timing of those activities were disrupted. She became disoriented by that. So that’s when I knew that Namaste Care had an impact on how she is. (CG-205)

##### The program instilled confidence in persons living with moderate dementia to participate in different activities

Caregivers perceived that the program provided opportunities to support persons with moderate dementia in completing as many activities as they could. Caregivers of persons with moderate dementia felt it was important for their family members to retain their abilities as much as possible and encourage them to participate in different activities.

Well, I think it also gives her a little bit of confidence too, she is doing different things because when she is with my dad…he doesn’t let her do anything, so it takes her away from the boredom of just sitting there at the TV…I think she had some fun. So, she’s been enjoying music and she’s been exercising, so we get her a little bit in shape. And she is trying different food that my dad would have never have eaten… (CG-206)

Caregivers were present to assist persons with dementia even if activities were challenging at first such as completing a puzzle or baking.

#### Recommendations for further adaptations

Although caregivers were highly satisfied with the overall program, some had recommendations to improve the program. Themes were: (a) offering more caregiver training and information on how to involve others (e.g., other family members, personal support workers) in delivering the program; (b) further tailoring of items provided in the Namaste Care Toolbox to increase the likelihood of engagement; and (c) reformatting the Namaste Care activities checklist.

##### Offering more caregiver training and information on how to involve others (e.g., other family members, personal support workers) in delivering the program

Despite being provided with a training session and manual, there was still some uncertainty for caregivers in how they should be presenting items to persons with dementia and using them effectively. One caregiver recommended role playing during the training session to simulate how an activity may be delivered during a session to maximize the use of an item such as a doll.

Because I am new to this, I think even having examples of how to do it [activities]…I guess, it could build confidence if we almost role played…Because sometimes, I have something and my mom would say ‘what am I supposed to do with this?’ And I’ll be like ‘I am not entirely sure, but I think we could do with it…’ You know in a way, it could be good, because maybe we explore ways and ways that were not intended. But maybe they could be used better than what I had used them for. (CG-206)

On a few occasions, some caregivers engaged other family members to take part in learning and delivering the program together. Some family members joined caregivers in delivering the program and others were hesitant to do so. Some caregivers saw the value that this program could bring for personal support workers in the delivery of their care and interactions for persons with dementia. This finding reveals the need for greater support and information on how to involve others such as family members and personal support workers to use the program.

##### Further tailoring of items provided in the Namaste Care Toolbox to increase the likelihood of engagement

Given that one of the barriers to delivering the program consisted of persons with dementia not responding to all items included in the Toolbox, caregivers recommended further tailoring prior to sending the Toolbox. Although MY had a conversation with caregivers about the items to be included and whether they would be things that the person they were caring for may like to try, there could have been different options provided in case caregivers were unsure if activities were too hard or easy for individuals such as puzzles. *“Have a bigger piece of puzzle to begin with and maybe have a little piece puzzle like you did. But…depending on the person, maybe putting in a different kind of puzzle”* (CG-210). Some caregivers were hoping to deliver various types of activities such as arts and crafts and building small cars, however some caregivers felt that their family members had progressed beyond such activities throughout the 3-month study period. *“As he got worse, its just made it harder to do the other things that I was hoping to do. That were in the box”* (CG-204).

##### Reformatting the Namaste Care activities checklist

Although caregivers appreciated receiving a checklist to record activities completed during program sessions, some caregivers perceived that it was challenging to complete because it was organized by month and caregivers did not always start the program the first day of the month.

I don’t know how you can do it. But it’s sometimes confusing to have the first, first day of the week and all of sudden it skips to the middle of the week. You know, because I would kinda fill it in and start doing and realize, “Oh, today is not the 6^th^”. (CG-209)

They recommended having a space to record the day of the week or having the whole month on a single sheet. One caregiver recommended a fillable online checklist that could be completed on a hand-held device such as a phone.

### Preliminary effectiveness results

**Fig. 2:**
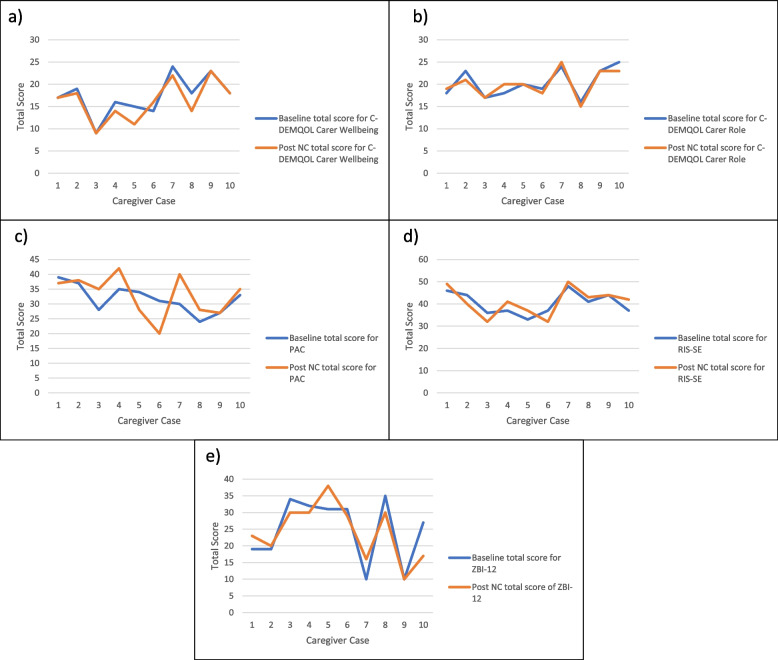
Scores at baseline and 3-month by caregivers. Legend: NC = Namaste Care; a) and b) QOL = quality of life, C-DEMQOL = Carers-DEMentia Quality of Life scale, Higher scores reflect better QOL; c) PAC = Positive Aspects of Caregiving scale, Higher scores reflect greater positive feelings towards caregiving; d) RIS-SE = Relational, Instrumental, Self-soothing Eldercare Self-Efficacy scale, Higher scores reflect greater self-efficacy; e) ZBI-12 = Short form Zarit Burden Interview scale, Higher values represent greater burden

This section describes the preliminary effects of the adapted Namaste Care program in terms of QOL, perceptions of caregiving, self-efficacy, and caregiver burden. See Fig. 2 for the baseline and 3-month scores for all outcomes. Despite some favorable changes in some outcomes, these were very small changes that are graphically shown on figures and none of the changes were statistically significant. For QOL of caregivers measured by two sub-domains of C-DEMQOL, a mean difference of -1.10 (95% CI: -2.47, 0.27; *p* = 0.102) was found for C-DEMQOL Carer Wellbeing and -0.20 (95% CI: -1.14, 0.74; *p* = 0.642) for C-DEMQOL Carer Role. A mean difference of 1.20 (95% CI: -3.36, 5.76; *p* = 0.566) for perceptions of caregiving and a mean difference of 0.70 (95% CI: -1.98, 3.38; *p* = 0.569) for self efficacy were found. A mean decrease of -0.50 (95% CI: -4.26, 3.26; *p* = 0.770) was found for caregiver burden. See Table 6 for the baseline and post-intervention preliminary effectiveness results.

**Table 6:** Baseline and post-adapted Namaste Care program preliminary effectiveness results (*N* = 10)

Outcome	Measure	Baseline Mean [SD]	Post-adapted Namaste Care program (3-months) Mean [SD]	Mean difference [95% CI]	*P*-value
Caregiver wellbeing QOL	**C-DEMQOL Carer Wellbeing**	17.30 [4.32]	16.20 [4.42]	-1.10 [-2.47, 0.27]	0.102
Caregiver role QOL	**C-DEMQOL Carer Role**	20.30 [3.20]	20.10 [3.04]	-0.20 [-1.14, 0.74]	0.642
Perceptions of caregiving	**PAC**	31.80 [4.69]	33.00 [6.94]	1.20 [-3.36, 5.76]	0.566
Self-efficacy	**RIS-SE**	40.30 [4.99]	41.00 [6.13]	0.70 [-1.98, 3.38]	0.569
Caregiver burden	**ZBI-12**	24.80 [9.59]	24.30 [8.53]	-0.50 [-4.26, 3.26]	0.770

*C-DEMQOL* Carers-DEMentia Quality of Life scale, *PAC* Positive Aspects of Caregiving scale, *RIS-SE* Relational, Instrumental, Self-soothing Eldercare Self-Efficacy scale, *ZBI-12* Short form Zarit Burden Interview scale, *CI* Confidence Interval

Multiple imputation results did not reveal any statistically significant changes from baseline to the 3-month follow-up outcome scores. A promising finding was however found when examining the individual imputations as 2 out of 5 imputations showed statistically significant increases for caregiver QOL in terms of caregiver wellbeing. For the 2^nd^ imputation for QOL in terms of caregiver wellbeing the mean difference was 1.08 (95% CI: 0.13, 2.04; *p* = 0.03). For the 3^rd^ imputation for QOL in terms of caregiver wellbeing the mean difference was 1.42 (95% CI: 0.19, 2.64; *p* = 0.03). See Table 7 for the multiple imputation results.

**Table 7:** Multiple imputation results

Outcome^a^	Imputation Number	Mean Difference	95% CI for Mean Difference	T test stat (*P*-value)	Pooled Results – Mean Difference [T test stat, p-value, 95% CI]
**Caregiver wellbeing QOL**	1	0.50	[-0.57, 1.57]	1.03 (0.32)	0.80 [1.16, 0.31, -1.13 – 2.73]
	2	1.08	[0.13, 2.04]	2.49 (0.03)*	
	3	1.42	[0.19, 2.64]	2.54 (0.03)*	
	4	0.42	[-0.74, 1.58]	0.79 (0.45)	
	5	0.58	[-0.52, 1.68]	1.17 (0.27)	
**Caregiver role QOL**	1	0.42	[-0.42, 1.25]	1.10 (0.29)	0.37 [0.96, 0.36, -0.50–1.23]
	2	0.25	[-0.52, 1.02]	0.71 (0.49)	
	3	0.33	[-0.49, 1.16]	0.88 (0.39)	
	4	0.42	[-0.42, 1.25]	-1.10 (0.29)	
	5	0.42	[-0.42, 1.25]	-1.10 (0.29)	
**Perceptions of caregiving**	1	-2.08	[-4.87, 0.70]	-1.65 (0.13)	-2.17 [-1.46, 0.18, -5.60 – 1.26]
	2	-2.33	[-5.82, 1.15]	-1.47 (0.17)	
	3	-2.92	[-6.00, 0.17]	-2.08 (0.06)	
	4	-1.75	[-4.68, 1.18]	-1.31 (0.22)	
	5	-1.75	[-4.68, 1.18]	-1.31 (0.22)	
**Self-efficacy**	1	-1.25	[-3.45, 0.95]	-1.25 (0.24)	-1.25 [-1.26, 0.24, -3.51 -1.01]
	2	-1.58	[-3.43, 0.26)	-1.89 (0.09)	
	3	-1.33	[-3.56, 0.89]	-1.32 (0.21)	
	4	-1.17	[-3.31, 0.98]	-1.20 (0.26)	
	5	-0.92	[-3.02, 1.19]	-0.96 (0.36)	
**Caregiver burden**	1	0.92	[-2.18, 4.01]	0.65 (0.53)	0.78 [0.52, 0.61. -2.60, 4.17]
	2	0.58	[-2.45, 3.62]	0.42 (0.68)	
	3	0.67	[-3.14, 4.47]	0.39 (0.71)	
	4	1.17	[-2.10, 4.34]	0.81 (0.44)	
	5	0.58	[-2.45, 3.62]	0.42 (0.68)	

*QOL* Quality of life

^*^ denotes a *p*-value of less than 0.05

^a^ Outcomes expressed as differences between baseline and 3-months

### Mixed methods interpretations

In this section we provide a summary of overall findings from Phase 2. We selected and adapted areas of focus and outcomes of interest from the Bowen et al. (2009) Feasibility Framework [39]. The categories were: (a) feasibility with regards to demand, implementation and practicality; (b) acceptability of the program; (c) limited preliminary effectiveness testing; and (d) further adaptations. See additional file 2 for the joint display of quantitative and qualitative findings.

#### Feasibility with regards to demand, implementation, and practicality

Evidence of the demand for the adapted Namaste Care program was its actual use and the positive perceptions of caregivers towards the program. With regards to degree of implementation, caregivers felt supported and confident in implementing a variety of different activities during a session due to the bi-weekly check-ins, information received, and receiving a Namaste Care Toolbox. More than half of the caregivers provided 7 out of 9 activities from the checklist over the 3-month period. Evidence of the program being manageable and seen as beneficial was it being delivered at least twice a week by caregivers over the intervention period. Caregivers perceived that barriers to program implementation included meeting other demands such as work and household tasks and persons with dementia not wanting to engage in activities at times. These barriers may be reflective as to why snacks and beverages and reminiscing activities were provided more often as these may require less time to implement and persons in moderate to advanced stages of dementia may be more willing to accept these activities. Based on the feasibility results, caregivers were able to consistently deliver the same number of activities throughout the 3-month study period indicating the likelihood that a routine for implementing activities had developed for caregivers.

#### Acceptability of the program

Caregivers were highly satisfied with using the program and the supports provided. Caregivers perceived that the virtual training session, written resources, bi-weekly check-ins, and the Namaste Care Toolbox were helpful in delivering the adapted Namaste Care program. Being satisfied with the program led to its frequent use. The program was perceived as appropriate for persons with dementia and caregivers felt comfortable in delivering it as well as trying different activities to engage their family members. All of the caregivers shared their interest in continuing to use the program after the study was completed.

#### Limited preliminary effectiveness testing

Quantitative results for caregiver QOL revealed that the program did not result in a statistically significantly improvement in caregivers’ QOL. This finding aligns with qualitative notes recorded during the bi-weekly check-ins where caregivers discussed the stress of caregiving and trying to adapt to deteriorating conditions of persons with dementia. Despite non statistically significant changes found for all outcomes, positive changes were seen for some outcomes such as perceptions of caregiving, self-efficacy, and caregiver burden. These changes converge with data obtained from qualitative interviews and check-ins as caregivers reported a greater awareness of the need for meaningful activities, perceived that the program brought them closer in their relationships with persons with dementia, and benefited them and persons with dementia. Caregivers did not perceive the program as creating additional burden as it was practical and manageable to deliver consistently.

#### Further adaptations

Although 66.7% of persons with dementia were in the moderate stages of dementia, activities such as arts and crafts and games were not delivered on a weekly basis and were delivered by only a few caregivers. This finding aligns with the suggestions received to increase supports for training and to provide more demonstrations of activities to increase the confidence of caregivers in delivering these. Further tailoring of items included in the Namaste Care Toolbox may also be required to ensure that the level of difficulty associated with specific arts and crafts and games are appropriate for each person with dementia. Persons with dementia may also benefit from multiple options for activities with varying levels of difficulty. Multiple options may also help persons with dementia as they transition to advanced stages and their capabilities and interests change.

## Discussion

To our knowledge this study is the first to implement and evaluate the adapted Namaste Care program for caregivers of older persons with moderate to advanced dementia at home in the community. The key findings of this present study were: (a) the adapted Namaste Care program and its proposed activities were feasible and acceptable and (b) caregivers perceived that the adapted Namaste Care program had many benefits for them and older persons with moderate to advanced dementia despite the lack of statistically significant differences in outcome measures.

Caregivers were able to deliver the adapted Namaste Care program at least twice a week for 3 months and the activities proposed were provided regularly on a weekly basis. The ability of caregivers to deliver the program and their interest in continuing to use the program after 3 months is also reflective of the support that they received consisting of regular contact with MY who is a Registered Nurse with experience in supporting dementia care. Support provided to caregivers can greatly alleviate caregiver burden related to changes in the cognitive function and mood of persons with dementia [89].

In terms of the activities delivered by caregivers, we conducted an analysis of the types of activities provided during the program sessions. Despite the growing research on Namaste Care, none of the studies to date described the frequency of use of various activities and none explored the activities delivered by family caregivers [30, 33–37]. Roland and Chappell (2015) explored the types of meaningful activities that persons with dementia engaged in based on the perspectives of family caregivers, however close to 60% had mild dementia and most of the activities were done outside the home such as social outings and playing sports [90]. The findings of the present study revealed that persons with moderate to advanced dementia can engage in simple, meaningful activities without leaving the home and without creating additional burden on caregivers.

Arts and crafts were less often offered for persons with dementia in the present study and this may be due to older persons with dementia being in advanced stages that limit their ability to engage in these types of activities. The activities included in Namaste Care such as aromatherapy, offering snacks and beverages, music, reminiscence, and touch/massage also have proven efficacy for persons with advanced dementia [33]. Hui et al. (2021) conducted a systematic review of psychosocial interventions for persons with moderate to severe dementia. These interventions however were not limited to home settings [23]. For persons with moderate to advanced dementia, aromatherapy and reminiscence had the strongest evidence in improving QOL and there was some evidence of improved cognitive function for a multicomponent program implemented in hospital involving music, exercise, and games [22, 91].

With regards to qualitative findings, caregivers perceived that the adapted Namaste Care program had many benefits for them and older persons with moderate to advanced dementia. Caregivers in the present study perceived that the program helped them to incorporate structure and creativity in engaging in activities with persons with dementia, improved their understanding of meeting the needs of their family members, and brought them closer in their relationships through mutual enjoyment of activities. Other studies exploring the experiences of family caregivers in participating in delivering Namaste Care in LTC similarly found that they formed improved connections with persons with dementia through the program and preferred delivering activities in which they shared personal interests such as games [30, 92].

Despite the positive perceptions caregivers had of the program in the current study and some promising improvement in scores, there were no statistically significant changes in caregiver QOL, perceptions of caregiving, self-efficacy, and burden, most likely due to the small sample size. Multiple imputation results did reveal a promising finding with 2 out of 5 imputations showing statistically significant increases for caregiver QOL in terms of caregiver wellbeing. This finding helps to shed light on the potential for a treatment effect by highlighting the inherent variation in the data. The research team perceived that it was important to include multiple imputation analyses for this feasibility study to explore the impact of missing data, as it is a common problem in studies on this population and one not often taken into account. We regard the results as exploratory since there has been little examination to date regarding the best methods for dealing with missing data in palliative care [76]. Mixed results have been seen for these outcomes in other studies of psychosocial interventions, which may reflect small samples, differences in program components or delivery mechanisms, choice of measures/outcomes, or the complexity of caring for persons with moderate to advanced dementia. To date only Lee (2021) used C-DEMQOL to evaluate caregiver QOL for a community singing program involving both persons with dementia and their caregivers delivered over six weeks [84]. However, only four caregivers completed their study due to COVID-19 restrictions which contributed to their lack of statistically significant results [84].

Another study including caregivers of persons with dementia that used the same perceptions of caregiving measure [70] used in our study found an improvement in perceptions of caregiving in the experimental (*n* = 13) versus control (*n* = 16) group following a memory and community program for family and persons with dementia dyads (*p* = 0.039) [26]. Czaja et al. (2013) similarly found a significant improvement in perceptions of caregiving following the implementation of a multicomponent psychosocial intervention for dementia caregivers delivered in-home and by videophone technology in the intervention (*n* = 26) versus control (*n* = 50) group (*p* = 0.006) [81]. The authors found that 46.2% of caregivers in the intervention group had improved perceptions of caregiving scores while in the control group only 16% had improved scores [81].

In terms of self-efficacy and caregiver burden, there is evidence of results similar to those found in this study for other studies of psychosocial interventions employing the same measures we used. For example, two studies showed no statistically significant changes in self-efficacy scores [80, 83] and three studies showed no statistically significant changes in caregiver burden scores [26, 80, 82]. These findings suggest that self-efficacy and caregiver burden are complex and may require a more targeted approach when implementing psychosocial interventions. They may also reflect challenges in managing the progressive nature of dementia and/or persons with moderate to advanced dementia.

Caregivers perceived that the adapted Namaste Care program had many benefits for persons with moderate to advanced dementia. These included ensuring that persons with dementia are engaged in meaningful activities consistently, enhancing the wellbeing of persons with dementia, and building the confidence of persons with moderate dementia to participate in different activities. There is growing evidence for the effectiveness of psychosocial interventions tailored to the specific needs of persons with dementia in increasing their wellbeing through decreased agitation and depression [23, 93]. Results from randomized controlled trials have shown that tailored activities for persons with dementia led to greater engagement, less agitation, and improved mood for persons with dementia as well as enhanced caregiver wellbeing and confidence in using activities [94, 95]. Ensuring that caregivers have the skills to deliver meaningful, tailored activities is also important to support their engagement in daily activities and social relationships [90]. These findings are in line with the adapted Namaste Care program and the delivery of tailored activities based on personalities and functional abilities [95].

The practice implication of this study is to work with community partners such as the Alzheimer Society to determine how best to offer the adapted Namaste Care program for caregivers. A possible option is to create an online toolkit made available through the Alzheimer Society website for caregivers to deliver the program and have a health or social care provider such as a nurse, social worker, or recreational therapist offer training and bi-weekly check-ins for caregivers to support them in delivering the program. As seen in the present study, caregivers valued the regular contact with a nurse in delivering the adapted Namaste Care program and caregivers may require this level of support to successfully deliver the program at home. Creating a peer support program can be helpful in connecting caregivers with experience in delivering the adapted Namaste Care program with caregivers who are new to the program. There is also a need to ensure that programs have room for flexibility so that they can be tailored to better meet the individual needs, abilities, and preferences of persons with dementia and caregivers.

The policy implication of this study is to offer education and support for psychosocial programs such as the adapted Namaste Care program within the community to build positive perceptions of caregiving and caregiver self-efficacy. There is a need to support organizations in offering evidenced-based interventions for their clientele to better meet their learning needs and support their caregiving practices. By supporting caregivers who care for persons with moderate to advanced dementia, persons with dementia can have their care needs met at home which can reduce the use of LTC and hospital services. Supporting caregivers to care for persons with dementia at home should be an important priority for the healthcare system due to long wait-times for LTC and the rising costs of LTC [15].

Despite the multitude of organizations that exist, caregivers continue to seek education and support for caring for older persons with dementia at home making this area an important priority for research [7, 96]. Future research is needed to evaluate the adapted Namaste Care program through a more rigorous research design such as a randomized controlled trial. Although caregivers were asked about benefits of the program for persons living with dementia during interviews and at the bi-weekly check-ins we did not use formal measures to assess preliminary effectiveness for persons with dementia. Some common outcomes that have been included in other studies evaluating Namaste Care, and that could be used in future studies include pain, QOL, depression, agitation, or medication use (e.g., antipsychotic or antidepressant medications) [58]. A next step could be to collect data on outcomes for persons with dementia receiving the adapted Namaste Care program at home such as QOL, pain, anxiety, and depression. A longitudinal study could be conducted. Caregivers could be asked about and receive training to complete questionnaires that measure these outcomes.

Future research should include sex and gender-based analysis as this was not examined in the present study. With regards to sex and gender considerations, male caregivers have been found to be more focused on task-based activities such as providing assistance with personal care compared to women [97]. Caregiving has often been regarded as female work [97]. For male caregivers the adapted Namaste Care program may have led to changes in behaviours and attitudes towards caregiving and supporting persons with dementia at home through meaningful activities. Female caregivers have been found to experience higher levels of caregiver burden compared to male caregivers [98, 99]. Pre-existing caregiver burden may have impacted the ability of female caregivers in this study to deliver the adapted Namaste Care program more frequently.

### Strength and limitations

The strengths of the study included the use of quantitative and qualitative methods to evaluate the implementation and preliminary effectiveness of the adapted Namaste Care program and the inclusion of caregivers from across Canada. Study limitations include weaknesses inherent in the before-after design, the absence of outcomes for older adults with moderate to advanced dementia, and the lack of ethnic or cultural diversity among caregivers. With a small sample size multiple imputation results should be interpreted with caution, and one should not be too quick to assume that no or limited significant changes were found with regards to caregiver outcomes. Strategies targeted at increasing the representation of Black, Hispanic, and Asian individuals in research are necessary as these populations experience high levels of discrimination in seeking dementia care and participating in research [100]. Sex and gender-based analysis in the study was also limited by the small sample size and lack of diversity among caregivers. It is possible that financial incentives such as provision of resources at no cost to participants and honorariums may have motivated participants to take part in the study. The use of convenience sampling may have led to selection and information bias as caregivers who participated in adapting Namaste Care were also included in the evaluation of the adapted program. Caregivers may therefore have formed prior relationships with MY.

## Conclusions

Overall findings of the present study revealed that the adapted Namaste Care program for use by caregivers of community-dwelling older persons with moderate to advanced dementia was feasible and acceptable. This is an important finding as caregivers supporting persons with dementia are highly involved in their day-to-day care, yet they were able to deliver the program without perceptions of increased burden. They found the program to be valuable and important to integrate into their caregiving routines. Despite the lack of statistically significant results supporting the preliminary effects of the program in terms of caregiver QOL, self-efficacy, positive perceptions of caregiving, and burden, caregivers perceived that the program had many benefits to support them in their caregiving role and enhanced the wellbeing of older persons with dementia. These positive findings warrant further investigation in future studies with a stronger design and larger sample size.

## Supplementary Information

**Additional file 1. **Namaste Care Activities Checklist.

**Additional file 2. **Joint display of quantitative and qualitative findings.

## Data Availability

The data for this research consists of questionnaires, interviews and transcriptions and notes. Raw data cannot be publicly released due to the risk of compromising participant confidentiality but are available from the corresponding author on reasonable request.

## Abbreviations

C-DEMQOL: Carers-DEMentia Quality of Life scale
LTC: Long-term care
MAR: Missing at random
PAC: Positive Aspects of Caregiving Scale
QOL: Quality of life
RIS-SE: Relational, Instrumental, Self-soothing Eldercare Self-Efficacy scale
ZBI-12: Short form Zarit Burden Interview scale
