# Supplementary material for: Feasibility, acceptability, and preliminary effectiveness of the adapted Namaste Care program delivered by caregivers of community-dwelling older persons with moderate to advanced dementia: a mixed methods feasibility study
Source: BMC Geriatr. 2022 Oct 13;22:797. doi: 10.1186/s12877-022-03483-9 (PMC9559259; doi:10.1186/s12877-022-03483-9)
Supplement: Supplementary file 1 — Additional file 1. Namaste Care Activities Checklist. [file 12877_2022_3483_MOESM1_ESM.docx]

**Additional File 1.** Namaste Care Activities Checklist

|  |  |  |
| --- | --- | --- |

**Study ID: CG -**

**Study Title:** Feasibility, Acceptability and Preliminary Effectiveness of the Adapted Namaste Care Program Delivered by Caregivers of Community-Dwelling Older Adults with Moderate to Advanced Dementia: A Mixed Methods Study

Please place a checkmark or a cross in the boxes when you have completed an activity for each day that you used the Namaste Care approach.

**D = Day**

| **Activity** | **D1** | **D2** | **D3** | **D4** | **D5** | **D6** | **D7** | **D8** | **D9** | **D10** | **D11** | **D12** | **D13** | **D14** | **D15** | **D16** |
| --- | --- | --- | --- | --- | --- | --- | --- | --- | --- | --- | --- | --- | --- | --- | --- | --- |
| Face washed/moisturized |  |  |  |  |  |  |  |  |  |  |  |  |  |  |  |  |
| Hands washed/moisturized |  |  |  |  |  |  |  |  |  |  |  |  |  |  |  |  |
| Feet washed/moisturized |  |  |  |  |  |  |  |  |  |  |  |  |  |  |  |  |
| Fingernails cleaned and clipped |  |  |  |  |  |  |  |  |  |  |  |  |  |  |  |  |
| Hair brushed |  |  |  |  |  |  |  |  |  |  |  |  |  |  |  |  |
| Offered a snack |  |  |  |  |  |  |  |  |  |  |  |  |  |  |  |  |
| Offered a beverage |  |  |  |  |  |  |  |  |  |  |  |  |  |  |  |  |
| Performed Range of Motion/Exercise |  |  |  |  |  |  |  |  |  |  |  |  |  |  |  |  |
| Reading |  |  |  |  |  |  |  |  |  |  |  |  |  |  |  |  |
| Aromatherapy/Scents |  |  |  |  |  |  |  |  |  |  |  |  |  |  |  |  |
| Family/friend visit |  |  |  |  |  |  |  |  |  |  |  |  |  |  |  |  |
| Played music |  |  |  |  |  |  |  |  |  |  |  |  |  |  |  |  |
| Telling stories/having conversations |  |  |  |  |  |  |  |  |  |  |  |  |  |  |  |  |
| Looking at photo albums or family videos |  |  |  |  |  |  |  |  |  |  |  |  |  |  |  |  |
| Providing a massage |  |  |  |  |  |  |  |  |  |  |  |  |  |  |  |  |
| Other: _____________ |  |  |  |  |  |  |  |  |  |  |  |  |  |  |  |  |
| Other: ________________ |  |  |  |  |  |  |  |  |  |  |  |  |  |  |  |  |
| Other: ________________ |  |  |  |  |  |  |  |  |  |  |  |  |  |  |  |  |
| **Activity** | **D17** | **D18** | **D19** | **D20** | **D21** | **D22** | **D23** | **D24** | **D25** | **D26** | **D27** | **D28** | **D29** | **D30** | **D31** |  |
| Face washed and moisturized |  |  |  |  |  |  |  |  |  |  |  |  |  |  |  |  |
| Hands washed and moisturized |  |  |  |  |  |  |  |  |  |  |  |  |  |  |  |  |
| Feet washed and moisturized |  |  |  |  |  |  |  |  |  |  |  |  |  |  |  |  |
| Fingernails cleaned and clipped |  |  |  |  |  |  |  |  |  |  |  |  |  |  |  |  |
| Hair brushed |  |  |  |  |  |  |  |  |  |  |  |  |  |  |  |  |
| Offered a snack |  |  |  |  |  |  |  |  |  |  |  |  |  |  |  |  |
| Offered a beverage |  |  |  |  |  |  |  |  |  |  |  |  |  |  |  |  |
| Performed Range of Motion/Exercise |  |  |  |  |  |  |  |  |  |  |  |  |  |  |  |  |
| Reading |  |  |  |  |  |  |  |  |  |  |  |  |  |  |  |  |
| Aromatherapy/Scents |  |  |  |  |  |  |  |  |  |  |  |  |  |  |  |  |
| Family/friend visit |  |  |  |  |  |  |  |  |  |  |  |  |  |  |  |  |
| Played music |  |  |  |  |  |  |  |  |  |  |  |  |  |  |  |  |
| Telling stories/having conversations |  |  |  |  |  |  |  |  |  |  |  |  |  |  |  |  |
| Looking at photo albums or family videos |  |  |  |  |  |  |  |  |  |  |  |  |  |  |  |  |
| Providing a massage |  |  |  |  |  |  |  |  |  |  |  |  |  |  |  |  |
| Other: _____________ |  |  |  |  |  |  |  |  |  |  |  |  |  |  |  |  |
| Other: ______________ |  |  |  |  |  |  |  |  |  |  |  |  |  |  |  |  |
| Other: ________________ |  |  |  |  |  |  |  |  |  |  |  |  |  |  |  |  |

**Note.** The Namaste Care weekly log sheet was modified from Simard (2013).
