# Supplementary material for: Feasibility, acceptability, and preliminary effectiveness of the adapted Namaste Care program delivered by caregivers of community-dwelling older persons with moderate to advanced dementia: a mixed methods feasibility study
Source: BMC Geriatr. 2022 Oct 13;22:797. doi: 10.1186/s12877-022-03483-9 (PMC9559259; doi:10.1186/s12877-022-03483-9)
Supplement: Supplementary file 2 — Additional file 2. Joint display of quantitative and qualitative findings. [file 12877_2022_3483_MOESM2_ESM.docx]

**Additional File 2.** Joint display of quantitative and qualitative findings

| **Adapted area of focus and outcome of interest of the Bowen et al. (2009) Feasibility Framework** | **Quantitative** | | **Qualitative** | **Mixed Methods Interpretation** | |
| --- | --- | --- | --- | --- | --- |
|  | **Feasibility** | **Preliminary effectiveness** | **Acceptability, experiences, perceived benefits, and adaptation** | **Convergent** | **Divergent** |
| Feasibility with regards to demand, implementation, and practicality   - Actual use - Intent to use - Degree of implementation - Ability of caregivers to deliver program activities | -100% of caregivers delivered the program at least 2 out of 7 days per week for at least 8 out of 12 weeks.  -83.3% of caregivers had 3 months of data collection completed (1 caregiver withdrew due to the death of the person living with dementia and 1 caregiver withdrew due to the persons’ living with dementia declining health)  - No safety or adverse effects reported related to program use.  -On average 10 (83.3%) of the caregivers offered **snacks and beverages** weekly for about 5 times a week.  -On average 9 (75%) of the caregivers delivered **reminiscing activities** weekly for about 4 times a week.  -On average 8 (66.7%) of the caregivers took part in **physical touch activities** weekly for about 3 times a week.  -On average 8 (66.7%) of the caregivers provided **audio/visual activities** weekly for about twice a week.  -On average 8 (66.7%) of the caregivers delivered **ROM/Exercise** weekly for about 3 times a week.  -On average 7 (58.3%) of the caregivers had **family/friend visits and/or outings** weekly for about twice a week.  -On average 7 (58.3%) of the caregivers delivered **comforting activities** weekly for about 3 times per week.  -On average 3 (25%) of the caregivers played **games** weekly for about once a week.  -On average 1 (0.8%) of the caregivers delivered **arts and crafts activities** weekly for about once a week. |  |  | **Convergent with acceptability and experiences findings:**  -Some activities were provided more frequently by caregivers such as snacks and beverages and reminiscing activities. A perceived barrier to delivering the program was having to meet numerous demands while caregiving. This may be reflective of caregivers choosing to provide activities that take less time to implement.  -Perceived barriers to program delivery such as persons with dementia not wanting to engage may have limited the ability of caregivers to provide a variety of different activities more frequently.  -More than half of caregivers regularly provided at least 7 out of 9 activities included in the Namaste Care checklist. Caregivers also perceived that the program fits well with their routines and was manageable.  -Caregivers perceived that being provided with information, bi-weekly-check-ins, and a toolbox of activities made it easy to deliver the program. This finding was supported by the feasibility results as caregivers were able to implement the program consistently and provided a variety of activities. |  |
| Acceptability of the program   - Satisfaction and experiences - Intent to continue use - Perceived appropriateness |  |  | - Caregivers were highly satisfied with the program as they felt supported in delivering activities through the bi-weekly check-ins, training resources, and Namaste Care Toolbox.  -All caregivers stated that they would continue to use the program as it was manageable and had many perceived benefits.  -Facilitators to program delivery were being provided with a Namaste Care Toolbox,  having written resources readily available and delivering activities in a language familiar to persons living with dementia.  -Barriers to program delivery were that persons living with dementia did not want to engage in activities at times, did not respond to all items provided in the Namaste Care Toolbox, and caregivers had to meet the numerous daily demands (e.g., work, housework, appointments).  - Delivering the program was perceived by caregivers as fitting into their routines.  -Bi-weekly check-ins provided opportunities for caregivers to receive validation, ask questions, and stay focused with the program. | **Convergent with feasibility results:**  -Caregivers expressed high satisfaction with all facets of the program including training, bi-weekly check-ins, and the Namaste Care Toolbox. Their satisfaction with the program was reflected in the feasibility results in terms of actual use.  -All caregivers expressed their intent to continue to use the program after the study was completed which would likely occur based on the feasibility results that revealed a variety of activities were delivery regularly per week. |  |
| Limited preliminary effectiveness testing   - Effects of the program for caregivers and persons living with dementia - Evidence of trends in changes for caregivers’ QOL, perceptions on positive aspects of caregiving, self-efficacy, and burden |  | **-C-DEMQOL-Carer Wellbeing:** Baseline mean of 17.30 [SD=4.32] and median of 17.50; 3-month post-program mean of 16.20 [SD=4.42] and median of 16.50; mean difference of -1.10 (95% CI: -2.47, 0.27; p=0.102).  **-C-DEMQOL-Carer Role:** Baseline mean of 20.30 [SD=3.20] and median of 19.5; 3-month post-program mean of 20.10 [SD=3.04] and median of 20.00; mean difference of -0.20 (95% CI: -1.14, 0.74; p=0.642).  -**PAC:** Baseline mean of 31.80 [SD=4.69] and median of 32.00; 3-month post-program mean of 33.00 [SD=6.94] and median of 35.00; mean difference of 1.20 (95% CI: -3.36, 5.76; p=0.566).  -**RIS-SE:** Baseline mean of 40.30 [SD=4.99] and median of 39.00; 3-month post-program mean of 41.5 [SD=6.13] and median of 41.50; mean difference of 0.70 (95%CI: -1.98, 3.38; p=0.569).  -**ZBI-12:** Baseline mean of 24.80 [SD=9.59]; 3-month post-program mean of 24.30 [SD=8.53]; mean difference of -0.50 (95% CI: -4.26, 3.26; p=0.770). | -The program encouraged caregivers to take on a more structured approach and use creativity in delivering meaningful activities.  -Caregivers gained a better understanding of the various needs (e.g., physical, emotional, social) of persons living with dementia and how to address these.  -The program brought caregivers and persons living with dementia closer in their relationships through mutual enjoyment of activities.  -Persons living with dementia were more engaged in meaningful activities consistently through the program.  -The program enhanced wellbeing of persons living with dementia by decreasing agitation, increasing verbal communication, and supporting their enjoyment of activities.  -For persons living with moderate dementia the program instilled confidence to participate in different activities. | **Convergent with acceptability, experiences, and perceived benefits:**  -Caregivers did not perceive that their QOL improved through the program. Scores for caregivers in terms of carer wellbeing and role mostly remained unchanged or worsened.  -There was a positive, yet nonsignificant increase in positive aspects of caregiving scores. Caregivers perceived that the program brought them closer with persons living with dementia through mutual enjoyment of activities.  -There was a positive, yet non significant increase in self-efficacy scores for caregivers. Caregivers perceived that the program increased their knowledge in providing a structured approach to activities and delivering these by being creative. They also gained an increased awareness and skills in how to meet the various needs of persons living with dementia.  -There was a non-significant decrease in caregiver burden scores indicating a positive improvement. Caregivers perceived the program to fit into their routines without being considered as extra work. | **Divergent with acceptability and perceived benefits:**  -No statistically significant results were found for changes to perceptions of caregiving, self-efficacy, and burden for caregivers. Some of these findings conflict with qualitative data as caregivers perceived they had more confidence to deliver activities. |
| Further adaptations   - Need for further changes to the program |  |  | -Caregivers perceived the need to reformat the Namaste Care activities checklist to make it easier to complete.  -Caregivers suggested further tailoring of items provided in the Namaste Care Toolbox to increase the likelihood of engagement.  -Caregivers perceived the need to offer more support and training for caregivers and information on how to involve others in the circle of care (e.g., other family members, personal support workers) in taking part in delivering the program. | **Convergent with feasibility results:**  -Some activities such as arts and crafts and games were delivered by few caregivers on a weekly basis although 66.7% of persons with dementia were in the moderate stages. This finding is aligned with the need for greater supports and training for caregivers in delivering these activities and further tailoring of items included in the Namaste Care Toolbox. |  |

**Note***.* QOL = Quality of Life; ROM = Range of Motion; C-DEMQOL = Carers-DEMentia Quality of Life scale; PAC = Positive Aspects of Caregiving scale; RIS-SE = Relational, Instrumental, Self-soothing Eldercare Self-Efficacy scale; ZBI-12 = Short form Zarit Burden Interview scale; CI=Confidence Interval; p = p-value.
